# Targeting sickle cell pathobiology and pain with novel transdermal curcumin

**DOI:** 10.1093/pnasnexus/pgaf053

**Published:** 2025-02-13

**Authors:** Yugal Goel, Mya A Arellano, Raghda T Fouda, Natalie R Garcia, Reina A Lomeli, Daniel Kerr, Donovan A Argueta, Mihir Gupta, Graham J Velasco, Richard Prince, Probal Banerjee, Sirsendu Jana, Abdu I Alayash, Joel M Friedman, Kalpna Gupta

**Affiliations:** Hematology/Oncology Division, Department of Medicine, University of California, Irvine, CA 92697, USA; Hematology/Oncology Division, Department of Medicine, University of California, Irvine, CA 92697, USA; Hematology/Oncology Division, Department of Medicine, University of California, Irvine, CA 92697, USA; Hematology/Oncology Division, Department of Medicine, University of California, Irvine, CA 92697, USA; Hematology/Oncology Division, Department of Medicine, University of California, Irvine, CA 92697, USA; Department of Chemistry and Center for Developmental Neuroscience, The College of Staten Island (CUNY), Staten Island, NY 10314, USA; Hematology/Oncology Division, Department of Medicine, University of California, Irvine, CA 92697, USA; Department of Neurosurgery, Yale School of Medicine, New Haven, CT 06520, USA; Pathology Department, VA Long Beach Medical Center, Long Beach, CA 90822, USA; Vascarta, Inc., Summit, NJ 07446, USA; Department of Chemistry and Center for Developmental Neuroscience, The College of Staten Island (CUNY), Staten Island, NY 10314, USA; Laboratory of Biochemistry and Vascular Biology, Center for Biologics Evaluation and Research, Food and Drug Administration, Silver Spring, MD 20903, USA; Laboratory of Biochemistry and Vascular Biology, Center for Biologics Evaluation and Research, Food and Drug Administration, Silver Spring, MD 20903, USA; Vascarta, Inc., Summit, NJ 07446, USA; Department of Microbiology and Immunology, Albert Einstein College of Medicine, Bronx, NY 10461, USA; Hematology/Oncology Division, Department of Medicine, University of California, Irvine, CA 92697, USA; Division of Hematology, Oncology and Transplantation, Department of Medicine, University of Minnesota, Minneapolis, MN 55455, USA

**Keywords:** curcumin, inflammation, pain, red blood cell, sickle cell disease

## Abstract

Several comorbidities of sickle cell disease (SCD) originate from red blood cell (RBC) instability, chronic inflammation, and oxidative stress. Development of scalable, cost-effective therapeutics suitable for chronic administration to prevent, attenuate, and perhaps reverse the consequences of RBC instability is needed. Curcumin has many of these attributes as a safe compound with antisickling, antiinflammatory, and antioxidant properties, but its translational potential has been constrained due to limited bioavailability from oral administration. The present study demonstrates the rapid and high bioavailability of a novel topical/transdermal (TD) curcumin gel formulation in the plasma and blood cells and its effectiveness in humanized sickle cell mice in: (i) ameliorating features of sickle cell pain hypersensitivity and axonal injury; (ii) reducing multiple manifestations of RBC instability including evidence of decreased hemolysis (reduced lactate dehydrogenase levels), enhanced RBC ATP levels along with decreased oxidative damage; (iii) decreasing multiple proinflammatory cytokines including interleukin-6, monocyte chemoattractant protein-1, granulocyte–macrophage colony-stimulating factor, and activation, normal T cell expressed and secreted protein in skin secretome; and (iv) reducing mast cell degranulation and activation. Our data suggest that an easy-to-use novel TD curcumin gel formulation has the potential to ameliorate chronic pain, improve RBC stability, and reduce inflammatory consequences of SCD.

**Significance Statement**

Sickle cell disease (SCD) is caused by a point mutation in the globin gene leading to sickling of red blood cells (RBCs). SCD is characterized by severe pain, inflammation, oxidative stress, and organ damage, which contribute to the poor quality of life and reduce survival. Therapies approved recently for SCD do not cure pain. Patients are often on multiple drugs which may have further side effects. We demonstrate that a novel topical/transdermal curcumin gel ameliorates chronic mechanical and cold pain hypersensitivity and significantly reduces axonal injury, inflammation, mast cell activation, and hemolysis and stabilizes RBCs in humanized sickle mice. Our findings provide a proof of principle for targeting sickle cell pain with a novel cost-effective and easily applicable transdermal curcumin.

## Introduction

Severe pain is common in sickle cell disease (SCD), frequently requiring chronic opioid therapy (1). SCD pathobiology involves hemolysis, oxidative stress, inflammation, neuroinflammation, hypoxia/reperfusion injury, and persistent peripheral and central nervous system (CNS) activation (2, 3). Persistent nociceptor activation and opioid therapy can contribute to the generation and maintenance of pain via central sensitization (4, 5). Therapies targeting the drivers of SCD pain are urgently needed.

Curcumin (1,7-bis-(4-hydroxy-3-methoxyphenyl)-1,6-heptadiene-3,5-dione) improves many pathophysiological states involving oxidative stress, inflammation, neuropathy, ischemia/reperfusion injury, vascular dysfunction, neurodegeneration, and pain (6). Our earlier studies revealed that using high-dose oral curcumin inhibited spinal microglial and astrocyte activation; reduced oxidative stress and pain-related substance P (SP); and significantly reduced mechanical and thermal hypersensitivity in Berkeley (BERK) sickle mice over a 4-week period, suggesting modulation of central pain mechanisms (7). However, despite high oral dose, complete amelioration of cold hypersensitivity could not be achieved after 4 weeks of treatment. Cold hypersensitivity is one of the leading manifestations of SCD pain in human subjects and sickle cell mice (8–12). Thus, poor bioavailability of oral curcumin and/or peripheral mechanisms involving the vascular system and mast cells (MCs) may be involved in cold hypersensitivity. Curcumin's effects on peripheral mechanisms of sickle cell pain-related pathobiology involving the dorsal root ganglion (DRG) neurons are not known. Sensory neurons in the DRG are the primary target of peripheral noxious stimuli, which then transmit the action potentials to the spinal cord through their axonal processes. Degenerative axonal changes affecting the neurites are a cause of neuropathy leading to pain such as that observed in chemotherapy-induced peripheral neuropathy, where pain may persist even after discontinuation of chemotherapy (13). Similarly, pain in SCD may continue even after transformative gene and bone marrow-targeting therapies (14). Since chronic pain persists in SCD, it is likely that noxious sickle pathobiology triggered by hemolysis of sickle red blood cells (RBCs) leading to oxidative stress and inflammation portends axonal damage in DRG neurons. The effect of curcumin on RBC stability, MCs, and inflammation has not been examined in SCD.

Chronic administration of high doses of oral curcumin is required to achieve therapeutic efficacy, which is often modest. High chronic doses of oral curcumin can cause gastrointestinal disturbances in individuals on multiple medications to manage SCD (15). To overcome this limitation, we used a novel, stable, bioavailable, and easy-to-deploy topical/transdermal (TD) formulation of curcuminoids to examine the peripheral mechanisms involving inflammation and RBC stabilization underlying the antinociceptive effects of curcumin. We used humanized HbSS-BERK sickle mice expressing >99% human sickle hemoglobin (Hb) that display many clinical features analogous to those seen in human subjects with SCD (16, 17) and their congenic control HbAA-BERK mice expressing normal human HbA (16, 18).

## Results

### TD curcumin pharmacokinetics

**Fig. 1.**
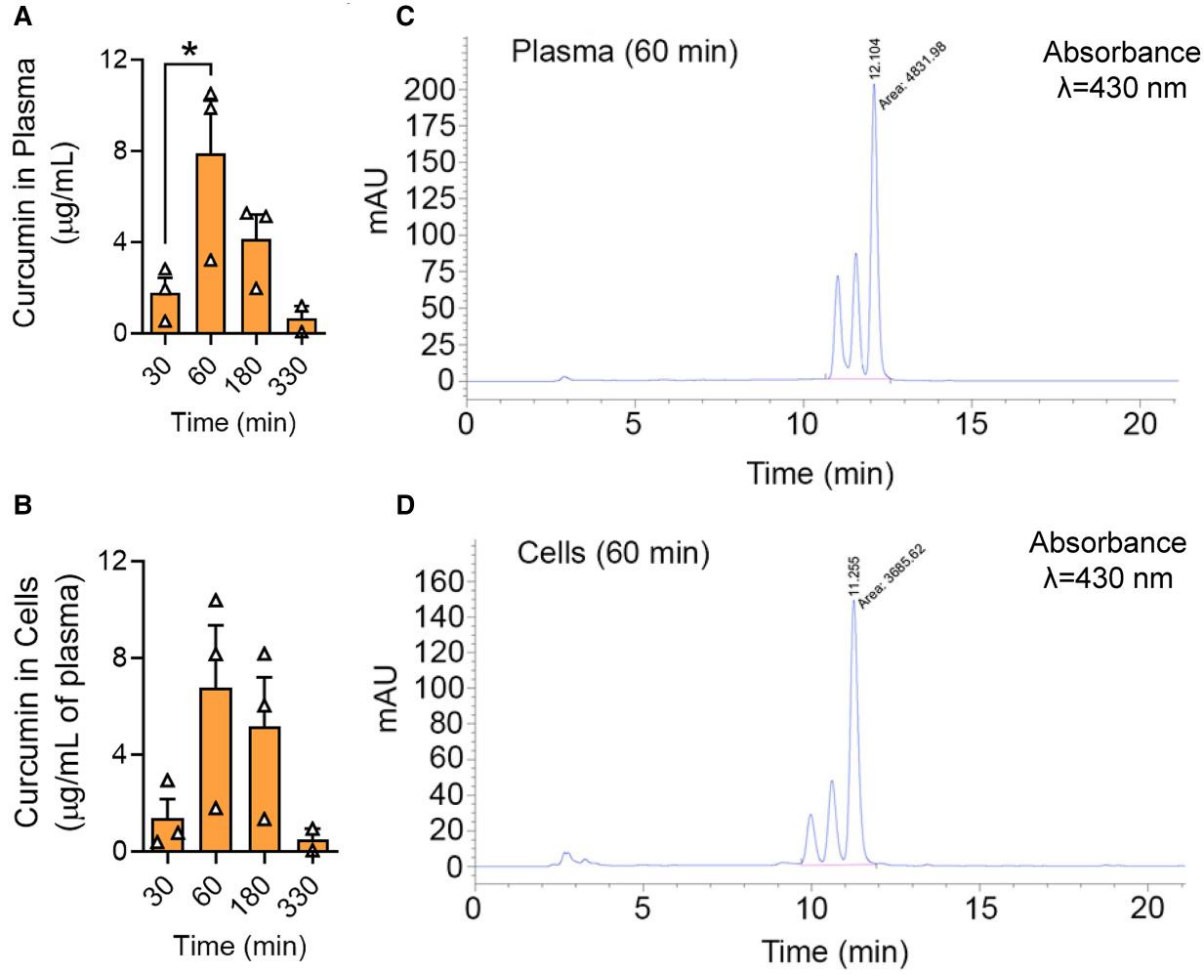
TD curcumin delivers curcumin in plasma and blood cells of C57BL/6 mice. A) Plasma curcumin levels are highest (mean 7.87 µg/mL) 60 min after topical application of Vasceptor. Plasma curcumin extracts are analyzed by HPLC. B) Curcumin levels in circulating cells are highest (mean 6.78 µg/mL) 60 min after topical application of Vasceptor. Curcumin in the cell pellet obtained after centrifugal separation of the plasma was extracted, and the extracts were analyzed by HPLC. The curcumin concentrations in cells are normalized to the volume of plasma and expressed as mg/mL. C) Representative HPLC chromatogram of mouse plasma collected 60 min following application of Vasceptor. The profile displays three peaks with areas of 980, 1,164, and 2,707 (AU) representing bisdemethoxycurcumin, demethoxycurcumin, and curcumin, respectively. The values in the *X*-axis represent elution time in minutes. D) Representative HPLC chromatogram of murine cells in circulation 60 min following application of Vasceptor. The profile displays three peaks with areas of 507, 799, and 2,441 (AU) representing bisdemethoxycurcumin, demethoxycurcumin, and curcumin, respectively. The values on the *X*-axis represent elution time in minutes. Data shown as mean ± SEM. *P < 0.05. Data analyzed with one-way ANOVA with Dunnett's multiple comparisons test. AU, arbitrary units; HPLC, high-performance liquid chromatography; TD, transdermal.

Pharmacokinetics results showed systemic delivery of TD curcumin. Following topical application of curcumin gel, plasma and total blood cell curcumin levels were evident within less than an hour. The levels in plasma and blood cells reached their peak concentrations (mean = 7.87 and 6.78 µg/mL, respectively) in C57BL/6J mice at 60 min posttopical treatment (Fig. 1A–D). These levels and systemic persistence are not achievable with oral dosing even when very high doses are given. These results show that: (i) TD curcumin results in sustained systemic bioavailability; (ii) plasma and cellular concentrations are proportional in mice; and (iii) TD curcumin likely overcomes the bioavailability limitations of oral curcumin.

### TD curcumin ameliorates pain-related behaviors

**Fig. 2.**
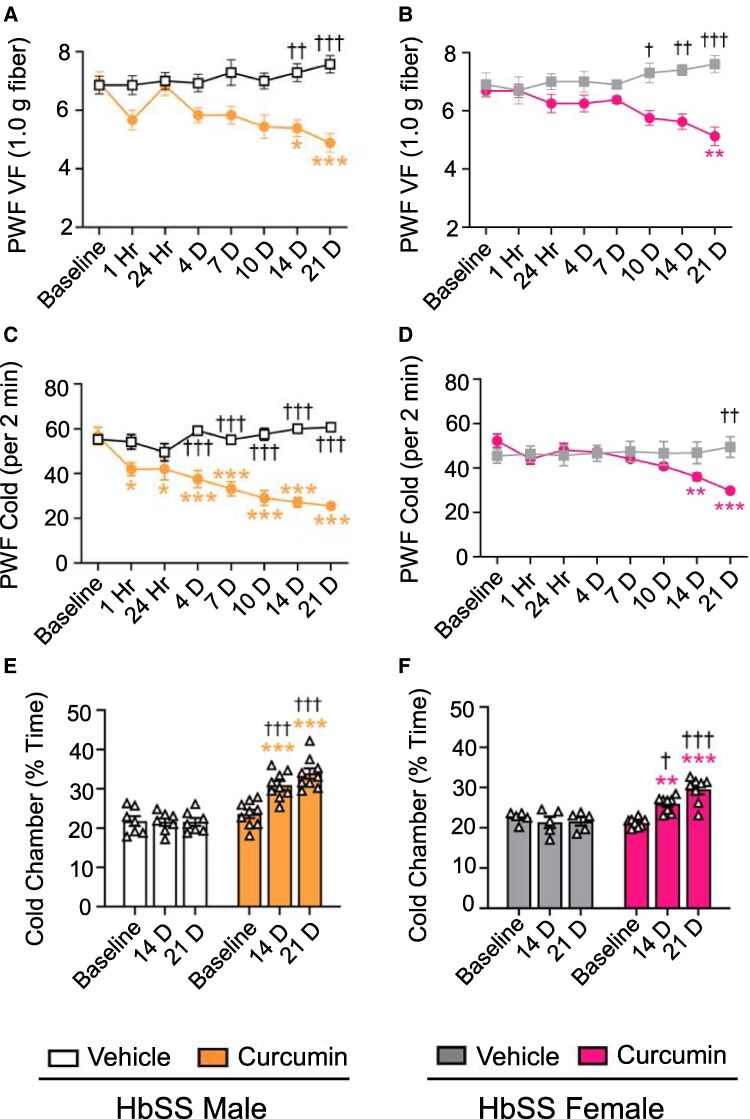
TD curcumin ameliorates cold and mechanical hypersensitivity in HbSS mice. A)–F) Male and female HbSS mice were treated with vehicle or TD curcumin for 3 weeks on alternate days by gently rubbing the gel on the abdomen. Behavioral measures were obtained at BL and on days 1, 4, 7, 10, 14, and 21 after start of treatment schedule. TD curcumin significantly improved A), B) mechanical and C), D) cold hypersensitivity indicated by lower PWF in response to von Frey monofilament (1.0 g fiber) application or 4 °C cold plate exposure, respectively. TD curcumin ameliorated E), F) nonevoked cold avoidance, indicated by increased time spent in a cold chamber. Data shown as mean ± SEM. Age: vehicle 5.40 ± 0.23 months and curcumin 5.37 ± 0.41 months. Data analyzed with two-way ANOVA with Tukey's multiple comparisons test. * indicates significance compared with curcumin BL. † indicates significance compared with vehicle at matching time point. *† *P* < 0.05; **†† *P* < 0.01; ***††† *P* < 0.001. *n* = 6–9. PWF, paw withdrawal frequency; TD, transdermal; VF, von Frey.

Pain-related behaviors were analyzed 1 and 24 h after initial dose or before treatment on following days as indicated with each figure and in Fig. S1. Main effects of treatment, time, and their interaction were significant for TD curcumin (Table S1). TD curcumin significantly decreased paw withdrawal frequency (PWF) in response to von Frey filament application, indicating reduced mechanical hypersensitivity from day 14 onward (Fig. 2A, *P* < 0.01 and *P* < 0.05 vs. vehicle and baseline [BL], respectively) and reduced PWF following cold plate (4 °C) exposure, corresponding with cold hypersensitivity (*P* < 0.05 vs. BL) from 1 h onward in male HbSS mice (Fig. 2C and Table S1), which may be due to the acute vasoregulatory effect of curcumin. TD curcumin reduced mechanical and cold hypersensitivity from days 10 and 21, respectively, vs. vehicle in female HbSS mice (Fig. 2B and D and Table S1, *P* < 0.05 and *P* < 0.01, for mechanical and cold, respectively). In a nonevoked cold avoidance test, TD curcumin significantly increased time spent in an aversive “cold” chamber from day 14 onward in male (*P* < 0.001 and *P* < 0.01 vs. vehicle and BL, respectively, Fig. 2E) and female HbSS mice (*P* < 0.05 and *P* < 0.01 vs. vehicle and BL, respectively, Fig. 2F). Female HbAA control mice did not show changes in pain-related hypersensitivity with TD curcumin (Fig. S2A–C). Musculoskeletal hypersensitivity did not improve in HbSS mice. This could be due to avascular necrosis, which may persist in ∼6-month-old HbSS mice used in this study (19). Similar to oral curcumin used in our previous study (7), TD curcumin significantly ameliorated mechanical hypersensitivity but had a greater effect on cold hypersensitivity in male HbSS mice. Delayed response of TD curcumin treatment to cold in female HbSS mice corroborates that females are more sensitive to cold than males due to several reasons including lesser muscle mass, lower metabolic rate, and fluctuating hormonal regulation with estrogen and progesterone compared with males; these factors contribute to altered constriction of the blood vessels in the extremities in response to cold or stress (20).

### TD curcumin rescues axonal degeneration of DRG neurons induced by a sickle microenvironment

**Fig. 3.**
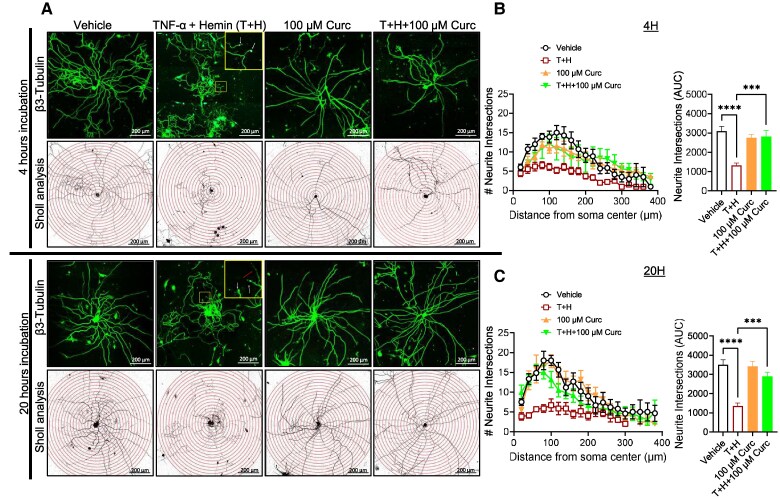
Curcumin inhibits TNF-α+hemin (T + H)-induced axonal degeneration in DRG neurons. A) DRG neurons from HbSS mice were exposed to 1 ng/mL of TNF-α and 40 μM of hemin with or without cotreatment of 100 µM of curcumin, or with 100 µM of curcumin alone, for 4 and 20 h. Representative images show neurites immunostained for β3-tubulin protein. The enlarged area in the inset indicates neurite swelling (retraction bulbs, white arrows) and discontinuous β3-tubulin protein expression with thinning of neurites (red arrow). The neurites are subsequently reconstructed using ImageJ software and superimposed over concentric Sholl circles spaced at 20-μm increments, providing a visual framework for analyzing the neuronal morphology and branching patterns. B), C) Neurite intersections at increasing distance from the soma and neurite intersections (AUC) for 4 and 20 h, respectively. Data shown as mean ± SEM. Sex: male. Age: 3.54 ± 0.41 months. Data analyzed by one-way repeated measures ANOVA with Tukey's post hoc analysis for multiple comparisons test. ***P < 0.001; ****P < 0.0001. *n* = 6–9 neurons/condition. Scale bars = 200 μm. AUC, area under curve; DRG, dorsal root ganglia; TNF-α, tumor necrosis factor alpha.

DRG neurons isolated from HbSS mice were maintained in primary culture for 24 h, followed by 4 and 20 h of incubation with vehicle or tumor necrosis factor alpha (TNF-α) + hemin (T + H) in the presence and absence of 100 µM curcumin. At both timepoints, vehicle-treated neurons showed exuberant neurite growth. Compared with vehicle, the neurites of DRG neurons in a sickle microenvironment with T + H for 4 and 20 h appeared disorganized and tortuous, with swollen peripheral neurite terminals that resembled “retraction bulbs,” which may lack a regenerative function (Fig. 3A, white arrows in the insets). Additionally at 20 h, discontinuous expression of the β3-tubulin protein in the neurites was observed with neurite thinning (red arrow in the inset). Further, the quantitative assessment of neurite arborization, measured by the number of neurite intersections at increasing distance from the soma, showed T + H exposure reduced the sum of neurite intersections by radial distance from the soma (area under curve) compared with vehicle treatment at 4 h (*P* < 0.0001) and 20 h (*P* < 0.0001), which was prevented by coincubation with curcumin compared with T + H at 4 h (*P* = 0.0003) and 20 h (*P* = 0.0006). Thus, TD curcumin may have a neuroprotective effect under a sickle microenvironment on primary DRG neurons involved in sensing and relaying the peripheral sensory inputs to the CNS. No significant change in the branching of neurites in the curcumin-alone-treated groups vs. vehicle was observed at either 4 or 20 h (Fig. 3B and C).

### Impact of TD curcumin on organs

**Fig. 4.**
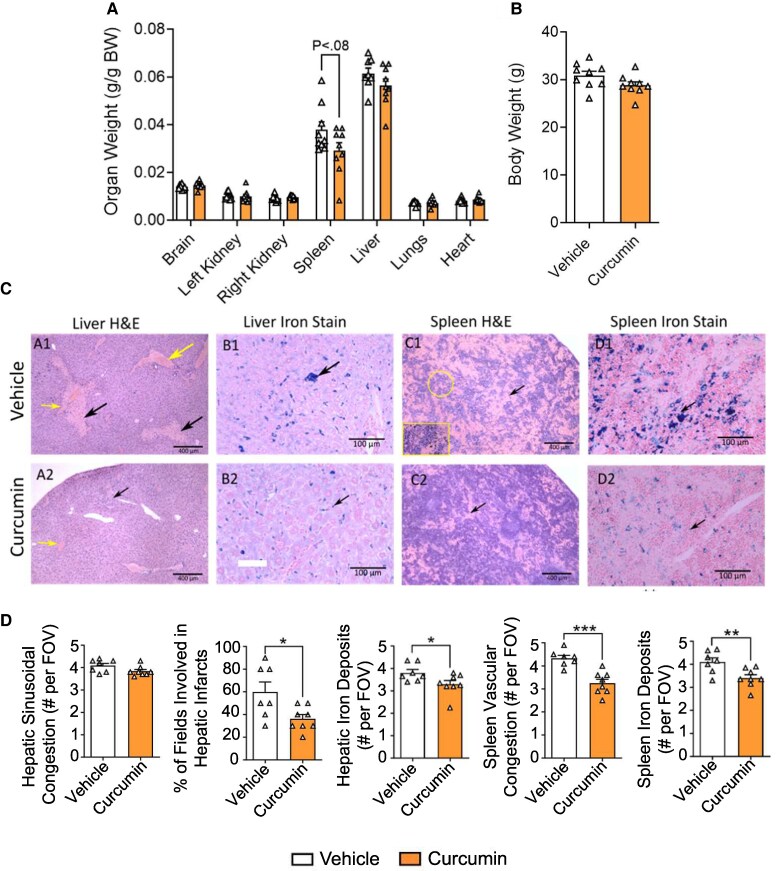
TD curcumin ameliorates vascular congestion and iron accumulation in liver and spleen of HbSS mice. TD curcumin treatment for 21 days A), B) did not adversely affect organ and body weights in male HbSS mice. TD curcumin C), D) showed a significant reduction in vascular congestion and iron deposits in sickle mice compared with vehicle in % fields involved in hepatic infarcts (black arrows), hepatic sinusoidal congestion (yellow arrows), splenic sinusoidal congestion (black arrows), and hepatic and splenic iron deposits observed with H&E and iron staining of formalin-fixed paraffin-embedded tissue. Data shown as mean ± SEM. Sex: male. Age: vehicle 5.40 ± 0.23 months and curcumin 5.37 ± 0.41 months. Data analyzed with unpaired Student's two-tailed t test. *P < 0.05; **P < 0.01; ***P < 0.001. *n* = 7–8. BW, body weight; H&E, hematoxylin and eosin; TD, transdermal.

TD curcumin had no significant effect on gross organ weights (brain, kidneys, liver, lungs, spleen, and heart) or body weights (Fig. 4A and B). TD-curcumin-treated HbSS mice showed reduced liver and spleen damage. The liver and spleen are traditionally recognized as primary sites of systemic iron storage where chronic hemolysis drives iron deposition in hepatic Kupffer cells and splenic macrophages (21, 22). The histopathology showed that TD-curcumin-treated HbSS mice exhibited a significant reduction in (i) the percentage of fields involved in hepatic infarcts and iron deposits in the liver and (ii) reduced vascular congestion and iron deposition in the spleen, as demonstrated by Prussian blue staining. Additionally, spleen architecture was better preserved with distinct red pulp and visible lymphoid follicles (white pulp), despite residual inflammation or hyperplasia (Fig. 4C and D). We observed increased macrophages in the TD-curcumin-treated spleens, which appears to be a reparative response as discussed in the Discussion section.

### TD curcumin improves hematological parameters

**Fig. 5.**
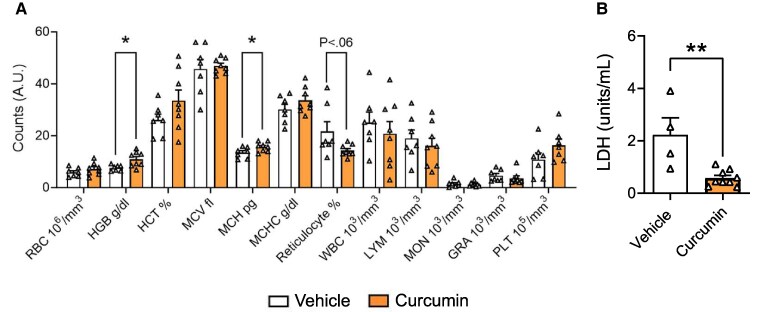
TD curcumin normalizes hematological parameters in HbSS mice. TD curcumin treatment for 21 days improved CBC A) hematological parameters in male HbSS mice as indicated by increased circulating Hb, increased MCH, and reduced reticulocytes. TD-curcumin-treated mice showed B) a reduction in plasma LDH, suggesting a reduction in hemolysis compared with vehicle treatment. Data shown as mean ± SEM. Sex: male. Age: vehicle 5.64 ± 0.89 months and curcumin 5.93 ± 1.13 months. Data analyzed with unpaired Student's two-tailed t test. *P < 0.05; **P < 0.01. *n* = 7–9. CBC, complete blood counts; GRA, granulocyte; HCT, hematocrit; Hb, hemoglobin; LDH, lactate dehydrogenase; LYM, lymphocyte; MCH, mean corpuscular hemoglobin; MCHC, mean corpuscular hemoglobin concentration; MCV, mean corpuscular volume; MON, monocyte; PLT, platelet; RBC, red blood cell; TD, transdermal; WBC, white blood cell.

Hematological parameters of the TD-curcumin-treated HbSS mice showed evidence of improvement compared with vehicle treatment (Fig. 5A). There were a significant increase in Hb (*P* < 0.05) and mean corpuscular Hb (*P* < 0.05) and a reduction in reticulocytes (*P* < 0.06) in whole blood of TD-curcumin-treated HbSS mice vs. vehicle. In SCD, lactate dehydrogenase (LDH) activity indicates hemolysis (23); TD curcumin significantly reduced plasma LDH activity (Fig. 5B, *P* < 0.01) in HbSS mice vs. vehicle, suggesting reduced hemolysis, which complements observed preventive effects of curcumin on RBC hemolysis as well as reduced iron deposits in the liver and spleen (24).

### TD curcumin improves the biochemical status of sickle RBCs from the treated mice

**Fig. 6.**
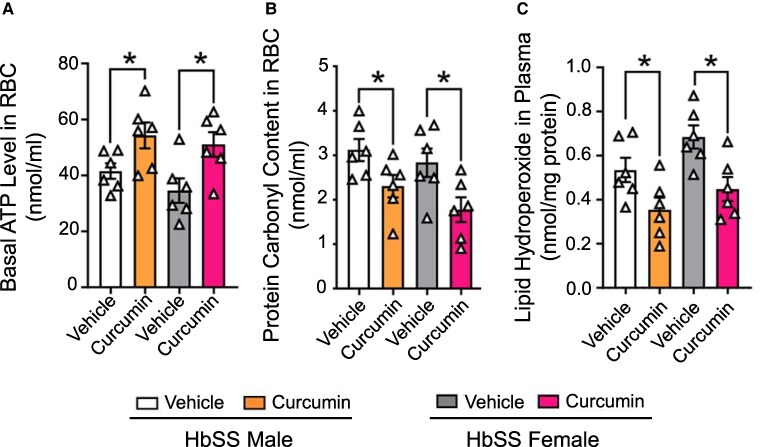
TD curcumin prevents ATP loss and reduces oxidative stress in HbSS mice. TD curcumin treatment of male and female HbSS mice for 21 days improved RBC energetics and oxidative stress. TD curcumin A) significantly increased RBC ATP concentrations in male and female HbSS mice. TD curcumin reduced B), C) markers of oxidative stress indicated by reduced protein carbonyl content in RBCs and plasmatic lipid hydroperoxide. Data shown as mean ± SEM. Sex: male. Age: vehicle 5.78 ± 1.41 months and TD curcumin 5.89 ± 1.52 months. Sex: female. Age: vehicle 6.13 ± 0.34 months and TD curcumin 6.11 ± 0.31 months. Data analyzed with unpaired Student's two-tailed t test. *P < 0.05. *n* = 6. ATP, adenosine triphosphate; RBC, red blood cell; TD, transdermal.

RBCs isolated from the treated HbSS mice displayed improvement in parameters associated with RBC health, stability, and pathogenicity (Fig. 6A–C). The RBCs from the treated HbSS mice showed enhanced levels of adenosine triphosphate (ATP) and decreased levels of oxidative damage to both proteins and lipids. ATP levels have been directly linked to RBC deformability, exoskeleton stability, and the ability of RBCs to promote vasoregulatory responses to sheer stress and hypoxia (25–28). Higher levels of protein and lipid oxidation in HbSS-BERK mice have been previously observed (29). Our data show a significant reduction in levels of RBC protein carbonyl and plasma lipid hydroperoxides in both TD-curcumin-treated male and female HbSS mice.

### TD curcumin reduces inflammation

**Fig. 7.**
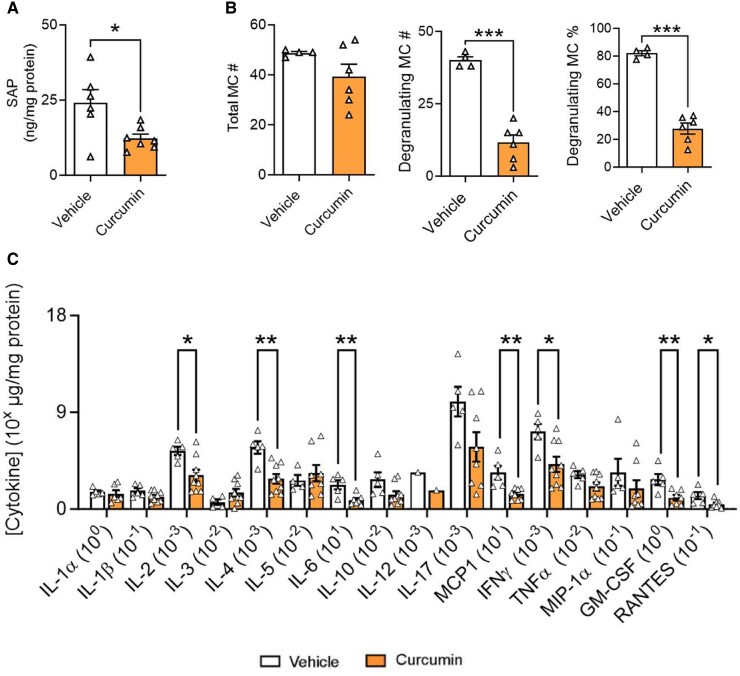
TD curcumin reduces inflammation in male HbSS mice. TD curcumin treatment for 21 days reduced markers of inflammation and cutaneous mast cell activation in male HbSS mice. HbSS mice treated with TD curcumin A) showed a significant reduction in global inflammation indicated by reduced concentration of circulating acute-phase SAP. Concomitant with reduced marker of global inflammation, TD curcumin B) reduced mast cell degranulation in the dorsal skin of HbSS mice stained with toluidine blue. TD curcumin C) significantly reduced markers of inflammation in skin biopsy-conditioned media indicated by reduced concentrations of pleiotropic IL-2, pleiotropic IL-4, mast cell-mediating IL-6, and proinflammatory MCP1, IFN-γ, GM-CSF, and RANTES compared with vehicle. Data shown as mean ± SEM. Age: vehicle 5.64 ± 0.89 months and curcumin 5.93 ± 1.13 months. Data analyzed with unpaired Student's two-tailed t test. *P < 0.05; **P < 0.01; ***P < 0.001. *n* = 7–9. GM-CSF, granulocyte–macrophage colony-stimulating factor; IFN-γ, interferon gamma; IL-1α, interleukin 1 alpha; IL-1β, interleukin 1 beta; IL-2, IL-3, IL-4, IL-5, & IL-6, interleukin 2–6; IL-10, interleukin 10; IL-12, interleukin 12; IL-17, interleukin 17; MC, mast cell; MCP1, monocyte chemoattractant protein 1; MIP-1α, macrophage inflammatory protein-1 alpha; RANTES, regulated on activation, normal T cell expressed and secreted protein; SAP, serum amyloid P; TD, transdermal; TNF-α, tumor necrosis factor alpha.

TD curcumin significantly reduced serum amyloid P (SAP; *P* < 0.05 vs. vehicle) a marker of global inflammation in HbSS mice compared with vehicle (Fig. 7A). Complementary to reduced inflammation, TD curcumin treatment led to decreased MC degranulation (Figs. 7B and S3). Further evidence of decreased inflammation in the TD-curcumin-treated HbSS mice is indicated by a significant reduction in multiple cytokines, including interleukin (IL)-2 (*P* < 0.05), IL-4 (*P* < 0.01), and IL-6 (*P* < 0.01), monocyte chemoattractant protein 1 (MCP-1; *P* < 0.01), interferon gamma (IFN-γ) (*P* < 0.05), granulocyte–macrophage colony-stimulating factor (GM-CSF; *P* < 0.01), and regulated on activation, normal T cell expressed and secreted protein (RANTES; *P* < 0.05), vs. vehicle in the skin secretome that are associated with the SCD state (Fig. 7C).

## Discussion

Herein, we provide the first evidence that TD curcumin ameliorates manifestations of chronic mechanical and cold hypersensitivity in both male and female HbSS mice. The high bioavailability of the topical application of the novel TD curcumin gel may be contributing to the beneficial effect of curcumin on RBC stabilization and reduction in inflammation alongside its antihyperalgesic effect.

The path to chronic pain is complex, but the root cause in SCD is likely RBC instability, which results in multiple consequences that can trigger pain-associated neuroinflammation. The decrease in damage to the spleen and liver is consistent with improved RBC stability. Most compelling with respect to RBC instability promoting chronic pain is the finding that hemolysis and other proinflammatory products from unstable RBCs both activate MCs (degranulating MCs) and endothelial cells through a toll-like receptor 4 pathway (30, 31). Findings suggest that tryptase released from degranulating MCs activates nociceptors via the protease-activated receptor-2, leading to the release of neuropeptides SP and calcitonin gene-related peptide, which increase vascular permeability, leading to neurogenic inflammation and pain hypersensitivity in HbSS mice (32). Inhibition of MCs with imatinib and genetic deletion of MCs in HbSS mice ameliorated thermal hypersensitivity (32).

The reduction in pain-like hypersensitivity and decrease in degranulating MCs can be accounted for through both a direct influence of curcumin on MCs and the improved hematological parameters that are consistent with enhanced RBC stability and less hemolysis. Less hemolysis may account for increased Hb and reduced reticulocytes, indicative of a disease-modifying effect.

The observed reduction in reticulocyte counts and improvements in hematocrit in TD-curcumin-treated SCD mice, while statistically significant, also represent biologically meaningful improvements within the context of the disease. These changes indicate a reduction in hemolysis and improved RBC stability, which are key therapeutic targets in SCD. Indeed, increasing hemolysis in Townes HbSS sickle mice suppressed erythropoiesis, suggesting the impairment in the ability of HbSS mice to compensate for anemia (33). It is likely that curcumin-induced increase in Hb is primarily due to the inhibition of RBC breakdown by stabilizing the RBCs. The increase in ATP and reduction in oxidative damage in the RBCs from the treated mice are supportive of enhanced stability/deformability and less hemolysis and microparticle formation (34–36). Furthermore, enhanced ATP levels correlate with sickle RBCs being less pathogenic with respect to vascular function, platelet activation, reduced endothelial NO production, and endothelial dysfunction (34–37). Eaton and colleagues observed through their phenotypic screen of the Scripps ReFrame repurposing library (38) that sickling is reduced 2-fold at a free concentration of 500 nM curcumin, similar to the reduction in sickling observed following gene addition therapy (39).

In an earlier study, TD curcumin limited endothelial dysfunction and preserved vascular integrity in a rat model of lipopolysaccharide-induced inflammation (40) and its antihematotoxic (41) and acute vasoregulatory (42) effects complement our observations of significant reduction in cold hypersensitivity and improvement in Hb-related parameters in HbSS mice. Our observations are supported by the antisickling effect of sickle erythrocytes from human HbSS sickle patients by ∼67% upon treatment with *Curcumin longa* rhizome extract (43).

Inflammation replete with “cytokine storm” is integral to SCD pathobiology and drives pain and central sensitization (44, 45). TD curcumin significantly reduced SAP (*P* < 0.05 vs. vehicle, Fig. 7A), a marker of global inflammation in mice analogous to acute-phase C-reactive protein in humans, which was complemented by reductions in multiple cytokines in the skin secretome. Significantly high GM-CSF, IL-2, IL-3, IL-4, IL-6, MCP-1, IFN-γ, and RANTES levels have been demonstrated in preclinical and clinical SCD (32, 46, 47). IL-6 and free heme are inducers of MCP-1 (48). Reduced hemolysis and increased Hb may be a novel pathway by which curcumin decreases MCP-1. In HbSS mice, MCP-1 and RANTES may contribute to chronic pain (32, 49) and MC activation (50). Earlier, we observed that GM-CSF correlates with MC degranulation in HbSS mice (32). Thus, activated MCs may remain active through autocrine signaling (51). By both improving RBC stabilization and reducing inflammation, TD curcumin may impart a neuroprotective effect on the nervous system.

DRG neurons in a sickle microenvironment in vitro with hemin and TNF-α treatment after 4 h show swelling of the tips of neurites similar to “retraction bulbs,” which may lack a regenerative function and significantly reduced neurite length. The axonal processes appear disorganized and tangled perhaps due to retraction and cytoskeletal disorganization instead of linear, well-organized axonal processes in vehicle-treated DRG neurons. Coincubation with curcumin prevents the swelling of axonal tips and normalizes axonal processes with significantly increased axonal length suggestive of a neuroprotective effect. Retraction bulbs may start forming within 1 h of injury and are an early manifestation of axonal degeneration (52). Axonal retraction occurs in peripheral neuropathies as “dying back” pathology with degradation of the axon from its tip (53), in degenerative conditions in addition to being induced by injury (Wallerian degeneration) (54). This is corroborated by our observations that axonal growth remains inhibited 20 h after incubation with T + H causing axonal stalling. In addition to many intracellular events including cytoskeletal organization, axonal growth requires energy from the mitochondria. Hemolysis is known to cause mitochondrial damage and deplete ATP in SCD (55). The observed increase of ATP in the RBCs of mice treated with TD curcumin supports the observations that TD curcumin treatment of DRG neurons inhibits retraction bulb formation and axonal stalling.

The blood–nerve barrier (BNB) maintains the integrity of the peripheral nervous system (56, 57). DRG is highly vascularized structures with increased vascular permeability compared with the vasculature in the brain (57, 58). Vascular endothelial growth factor (VEGF) and SP, which cause vascular permeability, are increased in mice and persons with SCD (59–63). VEGF has been shown to increase the permeability of BNB, which may drive neuropathic pain (64). VEGF with SP may increase vascular permeability in the DRG of HbSS mice, thereby allowing the infiltration of cell-free heme from hemolysis and proinflammatory cytokines into the DRG leading to axonal injury and hyperalgesia. Peripheral nerve injury led to the highest modulation of gene profiles of endothelial cells and pericytes in the DRG involving permeability- and angiogenesis-related genes (57). A brief report earlier demonstrated that tissue factor expression in pulmonary vein endothelial cells was significantly reduced by curcumin treatment in the NY1DD mouse model of SCD following hypoxia/reoxygenation challenge (65). We had earlier found that curcumin reduced spinal SP, which is known to cause vascular permeability in HbSS mice (7). The reduction in LDH, the enhanced stability of the RBCs following the treatment in vivo, the reduction in iron deposits, and blood flow stagnation in organs as well as multiple markers of inflammation (stemming from RBC instability) all support the conclusion that there is less hemolysis following TD curcumin. Oral curcumin has been shown to inhibit sickling in isolated human sickle RBCs from 49 subjects with SCD (43). We measured intraerythrocytic basal ATP levels in curcumin-treated HbSS mice to assess the metabolic/bioenergetic state of the RBCs. In both male and female mice, we found significant elevation (20–25%) of ATP levels in the treated groups compared with vehicle treatment. In addition to this, we also observed a profound antioxidant effect on macromolecules in TD curcumin treatment groups, e.g. a significant reduction of protein oxidation (protein carbonylation) and lipid peroxidation (lipid hydroperoxide formation). A higher level of oxidative markers associated with a higher level of heme oxygenase expression has also been reported in brain of Townes HbSS sickle mice, indicating a role of hemolysis and heme release in tissues of those sickle cell mice (29). Curcumin has been shown to attenuate iron accumulation in iron-overloaded rats and inhibit IL-6 (66, 67). Our histopathological observations on increased macrophages in the spleen of TD-curcumin-treated mice appear to be a compensatory response to clear excessive heme. Macrophages clear iron deposits, repair tissue damage, and remodel under iron overload and oxidative stress while maintaining immune defense. Curcumin promotes antiinflammatory M2 macrophage polarization, aiding tissue repair and resolving inflammation (68–70). The observed macrophages and organized spleen architecture with distinct white and red pulp reflect curcumin's reparative and antioxidative effects, supporting TD curcumin's therapeutic potential in improving spleen pathology in SCD mice. This is consistent with our observations of decreased iron deposits in the liver and spleen of HbSS mice. TD curcumin may therefore improve the noxious microenvironment in the DRG by reducing heme toxicity caused by hemolysis in HbSS mice leading to a reduction in hyperalgesia.

Together, our data show that a novel TD curcumin formulation significantly ameliorates hyperalgesia, hemolysis, MC activation, and inflammation and improves hematological parameters in HbSS mice. Thus, the lack of harmful effects, ease of topical curcumin administration, and its possible disease-modifying effects make it an attractive agent to evaluate further for its application in SCD.

## Materials and methods

### Mice

For examining the bioavailability of TD curcumin, we used ∼3-month-old male C57BL/6J mice (Jax, Bar Harbor, ME, USA). For behavioral and mechanisms underlying cold and mechanical hypersensitivity, we used ∼6-month-old humanized transgenic male and female homozygous HbSS-BERK “sickle” mice expressing >99% human sickle Hb, which show characteristic features of SCD pain, and female HbAA “control” mice expressing normal human HbA, without murine α- or β-globins (16, 18). The number of mice used is indicated with each figure. Mice were randomly assigned in a blinded manner and housed in standard 12-h light–dark conditions with *ad libitum* food and water. All animal experiments were performed after prior approvals from the UC Irvine (AUP-22-088) and VA Long Beach Healthcare System (ACORP 1618977) IACUC in accordance with guidelines from the National Institutes of Health (NIH).

### Treatment

We used a topical gel containing a proprietary, water-dispersible, bioavailability-enhanced formulation of curcuminoids (Curcugen, Dolcas-Biotech Inc.). Curcugen is dissolved in a PEG 400-based solvent with high solubility (0.1 M curcumin), stability, and deep skin permeation facilitated by addition of myristic acid in the gel. Mice were restrained by gently grasping the dorsal skin from the nape to expose the abdomen. We gently rubbed 0.1 mL of TD curcumin or inert gel (vehicle) to the abdomen every other day for 21 days (11 applications) as illustrated in the protocol schematic shown in Fig. S1.

### TD curcumin quantification in plasma and blood cells

Following TD curcumin application in C57B/L6 mice, blood was collected at different intervals between 0 and 330 min. Plasma and blood cells were separated, and curcumin was extracted from plasma and blood cells and analyzed using high-performance liquid chromatography (HPLC) on a C18 reverse-phase 4.6 × 250-mm column (Waters Corp, Milford, MA, USA) shown in Fig. S4 (71).

### Behavioral testing

HbSS and HbAA mice were acclimatized to the testing room and apparatus prior to testing, as previously described (8, 18). Pain-related behaviors were analyzed starting with mechanical, followed by musculoskeletal, and lastly cold hypersensitivity, with sufficient time between tests for rest. Behavioral measures were obtained at BL prior to administration of TD curcumin or vehicle, and posttreatment at different time intervals up to 21 days as indicated in Fig. S1. The 1- and 24-h recordings were performed after the first dose, and subsequent recordings were made prior to the delivery of the next dose.

### Isolation and culture of DRG neurons

The DRG from all spinal cord levels was removed and cultured as described previously (72). DRG neurons were dissociated with papain and collagenase/dispase into single-cell suspensions and plated onto poly-D-lysine-/laminin-coated glass coverslips.

### Beta III-tubulin immunostaining and analysis of neurites from DRG neurons

Primary DRG neurons were treated with either vehicle or 1 ng/mL TNF-α (T) + 40 µM hemin (H) in the absence or presence of 100 µM curcumin for 4 or 20 h at 37 °C. Cells were fixed with 2% paraformaldehyde (cat# 158127, Sigma-Aldrich, St. Louis, MO, USA) for 10 min at room temperature (RT) and permeabilized with ice-cold 0.1% Triton X-100 (Sigma-Aldrich) for 2 min, then blocked for 30 min with 3% donkey serum in PBS, and incubated overnight at 4 °C with primary antibody: rabbit β3-tubulin (Tuj1, 1:200, cat# 5568, Cell Signaling Technology, Danvers, MA, USA), followed by 1-h incubation at RT with secondary antibody: Cy3 AffiniPure donkey antirabbit IgG (H + L) (1:500, cat# 711-165-152, Jackson ImmunoResearch, West Grove, PA, USA) (73). Coverslips containing the cells were mounted onto glass microscope slides with ProLong diamond antifade mounting media (Thermo Fisher Scientific, Waltham, MA, USA) with nuclear counterstain 4′,6-diamidino-2-phenylindole. Appropriate negative controls for immunostaining were also used for DRG neurons. Images were acquired on a laser scanning confocal microscope (LSCM, Zeiss LSM 900, Carl Zeiss AG, Oberkochen, Germany), using a Plan-Apochromat 20x/NA: 0.8 M27 objective lens, using nine tile scans of 0.222 µm × 0.222 µm fields of view (arranged 3 × 3) with Z-stacks of 10 × 0.5-µm images. Images were processed for analyses by conversion into 8-bit monochromatic TIFF files, and concentric circles were overlaid on the images using the Concentric Circles plugin in FIJI (http://fiji.sc/Welcome, ImageJ, NIH, Bethesda, MD, USA). Sholl analysis was performed to quantify neurite branching using Simple Neurite Tracer plugin for FIJI (74). Starting at 20 μm from the center point of the soma, concentric circles were constructed 20 μm apart radiating from the soma. Neurites intersecting each concentric circle were enumerated and plotted against the radial distance from the center of the neuron's soma to calculate neurite branching.

### Body and organ weight

The body weights of mice were evaluated at BL and at day 21 of treatment. At the study endpoint (day 21), mice were euthanized in accordance with IACUC regulations using compressed medical-grade CO_2_. Blood and organs (brain, heart, lungs, spleen, kidneys, and liver) were collected immediately after euthanasia, and the organs were weighed and then fixed in 10% buffered formalin for paraffin embedding and subsequent analysis.

### Assessment of organ pathology

Liver and spleen sections (4 µm) were stained with standard hematoxylin and eosin (H&E) or commercial iron stain (AR15892, Agilent Technologies). Hepatic sinusoidal and splenic congestion (# per field), hepatic infarcts (% of fields), and hepatic and splenic iron deposits (# per field) were counted in 20 fields using four sections per animal (75).

### Analysis of hematological parameters

Whole blood was obtained by cardiac puncture following euthanasia with compressed CO_2_. Blood was combined with 100 mM EDTA pH 7.5 at a 2:1 ratio (blood/EDTA) and immediately analyzed for hematocrit, total Hb, and complete blood counts using Animal Blood Counter (abc Plus, Scilvet, Viernheim, Germany) and also stained with reticulocyte stain (cat# R4132, Sigma-Aldrich, Germany) to count the number of reticulocytes (32).

### LDH assay

LDH activity was measured in plasma samples by a colorimetric assay according to the manufacturer's instructions (MAK066, Sigma-Aldrich, St. Louis, MO, USA) (76). The absorbance was measured at 450 nm using a SpectraMax M3 plate reader (Molecular Devices, San Jose, CA, USA). Each specimen was run in duplicate with appropriate negative and positive controls. LDH activity is reported as milliunit/mL. One unit of LDH activity is defined as the amount of enzyme that catalyzes the conversion of lactate into pyruvate to generate 1.0 µmole of nicotinamide adenine dinucleotide per minute at 37 °C.

### Measurement of basal ATP level

Intracellular ATP levels in RBCs obtained after 21 days of curcumin or vehicle treatment from HbSS mice were measured using a colorimetric ATP assay kit (Sigma-Aldrich) as described (35). Briefly, RBCs were washed with PBS after collection, and then, 100 µL of packed RBCs was resuspended in PBS containing 1% glucose, 170 mg/L adenine, and 5 g/L mannitol for the ATP measurement. ATP concentration was determined by glycerol phosphorylation, which yields a colorimetric product proportional to the amount of ATP present, following the manufacturer's instruction. Absorbance was measured at 570 nm using a BioTek Synergy HTX microplate reader (Agilent, Santa Clara, CA, USA).

### Estimation of protein carbonylation and lipid hydroperoxide formation

As a measure of intracellular protein oxidation in frozen RBC lysates, protein carbonyl content was assessed by a dinitrophenylhydrazine (DNPH)-based assay kit (ab126287, Abcam, Cambridge, MA, USA) (77). In these experiments, carbonyl groups in protein side chains are derivatized to DNPH following reaction with DNPH. The absorbance of DNPHs formed in this reaction was measured at 375 nm using a BioTek Synergy HTX microplate reader (Agilent).

Lipid hydroperoxide levels were measured in plasma using a commercially available kit (705002, Cayman Chemical Co., Ann Arbor, MI, USA) (29). Briefly, lipid hydroperoxides were extracted from 500 µL of plasma using a methanol-based extraction solution and chloroform. Deproteinated methanol–chloroform extract solution containing lipid hydroperoxides was measured by incubating with a chromogenic substrate as indicated in the manufacturer's instructions. The absorbance was measured at 500 nm by a BioTek Synergy HTX microplate reader (Agilent). Extracts without chromogenic substrate were kept to be used as blanks to eliminate the possibility of curcumin interference.

### Estimation of SAP in plasma

SAP was estimated in plasma using an enzyme-linked immunosorbent assay (ELISA) as described by the manufacturer (80660; Crystal Chem, Elk Grove Village, IL, USA) and as described in (78). The absorbance for SAP assay was measured at 450 nm using a SpectraMax M3 plate reader (Molecular Devices). All analyses were run in duplicate with appropriate negative and positive controls as described previously (78).

### MC analysis

MCs in skin sections were stained with toluidine blue and recognized by red–purple metachromatic staining color on a blue background. MCs were counted in 20 fields, four sections per mouse, and expressed as total MC number, number of degranulating MCs, and percentage degranulating cells. Degranulating MCs were defined as cells associated with ≥ 8 granules outside the cell membrane, as described previously (32, 78).

### Cytokine array

Cytokines were quantified in skin releasate using a microplate-based Q-Plex microarray technology (Sample Testing Services of Quansys Biosciences Inc., Logan, UT, USA) as described in (32). The Q-Plex microarray uses traditional sandwich ELISA procedures on a microscale to simultaneously measure multiple cytokines/chemokines included in our study—inflammatory: IL-1 alpha, IL-1 beta, IL-2, IL-6, IL-12, IL-17, MCP-1, IFN-γ, TNF-α, macrophage inflammatory protein-1 alpha (MIP-1α), GM-CSF, and RANTES and antiinflammatory: IL-3–5 and IL-10. Total protein estimation in the conditioned medium was performed for normalization using the Pierce Protein Quantitation Assay (PI22662, Thermo Fisher Scientific).

### Statistics

Data are shown as mean ± SEM and analyzed with one-way or two-way ANOVA with Tukey's or Dunnett's multiple comparisons post hoc tests or unpaired two-tailed t test (Prism9, GraphPad, Boston, MA, USA). Comparisons with *P*-values <0.05 are considered statistically significant.

## Supplementary Material

pgaf053_Supplementary_Data

## Data Availability

All data are included in the manuscript and/or supporting information.
