# Supplementary material for: Targeting sickle cell pathobiology and pain with novel transdermal curcumin
Source: PNAS Nexus. 2025 Feb 13;4(2):pgaf053. doi: 10.1093/pnasnexus/pgaf053 (PMC11854080; doi:10.1093/pnasnexus/pgaf053)
Supplement: pgaf053_Supplementary_Data [file pgaf053_supplementary_data.docx]

**
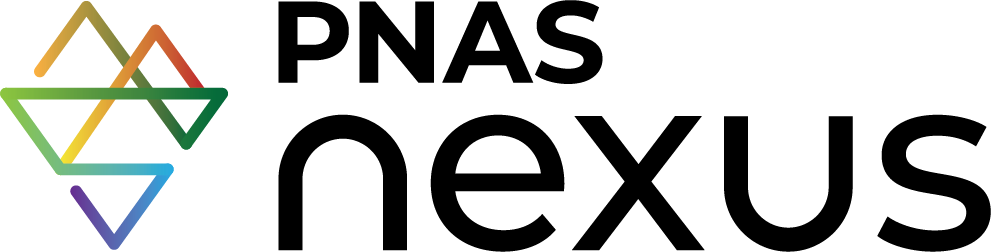
**

**Supplementary Information for**

Targeting sickle cell pathobiology and pain with novel transdermal curcumin.

Yugal Goel, Mya A. Arellano, Raghda T. Fouda, Natalie R. Garcia, Reina A. Lomeli, Daniel Kerr, Donovan A. Argueta, Mihir Gupta, Graham J. Velasco, Richard Prince, Probal Banerjee, Sirsendu Jana, Abdu I. Alayash, Joel Friedman, Kalpna Gupta^*^

*Kalpna Gupta

**Email:**  [kalpnag@hs.uci.edu](mailto:kalpnag@hs.uci.edu)

**This PDF file includes:**

Supplementary text

Figures S1 to S4

Tables S1

SI References 14

**Supplementary Information Text**

**Material and Methods**

**Mice**

To assess the bioavailability of a proprietary transdermal (TD) curcumin gel formulation after topical administration, we used healthy, ~3-month-old male C57BL/6J mice obtained from the Jackson Laboratory (Jax, Bar Harbor, ME, USA) and housed in the College of Staten Island Animal Care Facility. The mice were housed in a conventional 12-hour dark/light cycle, provided food and water *ad libitum,* and handled in accordance with CUNY- Institutional Animal Care and Use Committee (IACUC) approved procedures.

To evaluate the effect of TD curcumin on pain-like behaviors and biochemical measurements in sickle cell disease (SCD), we utilized ~6-month-old humanized male and female homozygous transgenic HbSS- Berkeley (BERK) ‘sickle’ mice expressing >99% human sickle hemoglobin (Hb), which show characteristic features of SCD pain.^1,2^ For control mice, we used HbAA-BERK mice expressing normal human HbA, without murine α- or β-globins on the same mixed genetic background of HbSS-BERK mice.^1,2^ Mice were bred by the Gupta laboratory and housed in a AAALAC approved facility as described.^2^ All pups were phenotyped, and those homozygous for sickle Hb were used, and those expressing only HbA without sickle Hb were used. Mice were randomly assigned to experimental groups in a blinded fashion. HbSS-BERK mice mimic the pathobiology and clinical features of pain in SCD, including mechanical, cold, and musculoskeletal hyperalgesia.^2-4^ All mice were housed in a conventional 12-hour dark/light cycle and provided with food and water *ad libitum*. All animal experiments were performed after prior approvals from the IACUC in accordance with guidelines from the National Institutes of Health (NIH).

**Treatment with TD curcumin gel formulation**

We used a novel biocompatible, topical non-aqueous proprietary gel formulation (Vascarta Inc, Summit, NJ, USA) containing a very high concentration of curcuminoids (0.1 M). The curcuminoids are provided through a proprietary turmeric root extract (Curcugen®, Dolcas-Biotech Inc, Landing, NJ, USA) containing high levels of all three naturally occurring curcuminoids. Curcugen® is dissolved in a polyethylene glycol 400 based solvent with myristic acid designed to promote high solubility, stability, and deep skin permeation. An experienced handler gently held the mice by gently grasping their dorsal skin from the nape to expose the abdomen. The formulation, an 8.5% curcuminoid topical/TD gel, and the proprietary vehicle (without Curcugen®) - both developed by Dr. Joel Friedman (Albert Einstein College of Medicine, Bronx, NY, USA) - were applied topically/transdermally by gently massaging 0.1 mL gel on the abdomen of mice with a foam swab for 2 minutes, every other day for 21 days (11 applications, Study Design, Suppl. Fig. S1).

**HPLC profiles from calibration curves for curcumin recorded using absorbance maximum at 430 nm**

A commercially available curcuminoid sample from Sigma containing 6% bis-demethoxy-curcumin, 17% mono-dimethoxy-curcumin, and 77% curcumin was dissolved in 70% Acetonitrile (ACN) in water to prepare a standard solution. The Standard solution was serially diluted to obtain solutions containing 5, 15, 25, 50, 75, 100, 125, 175, and 250 picomole of curcumin in 30 microliters of 70% ACN. Each solution was injected into a C18 HPLC column and eluted using a gradient of 30-90% ACN containing 0.1% TFA. The eluted curcuminoids were detected using absorbance at 430 nm.

Five sample eluate profiles of 5, 15, 25, 50, 75 picomole of curcuminoids are shown in the next slide. The areas under the three typical absorbance peaks in each eluate corresponding to 6% of bis-demethoxy-curcumin, 17% mono-dimethoxy-curcumin, and 77% curcumin are added and plotted against the pmole masses of curcuminoids to obtain a straight line (calibration curve). The profile obtained from 5 picomole of curcuminoids establishes the sensitivity of our method. The extracts of plasma and tissue samples containing curcuminoids are similarly eluted through the HPLC column, and the total area under the triplet peak obtained is compared with the calibration curve to obtain a quantitative measure of curcuminoids. (Figure S4)

**TD curcumin quantification in plasma and blood cells**

Mice were anesthetized and then treated topically (on a shaven belly) with 100 µL of the proprietary TD curcumin gel formulation. Mice were then placed under deep anesthesia by an intraperitoneal injection of 2.3 µL/g (~60-80 µL/mouse) ketamine/xylazine [mixture of 540 mL ketamine (50 mg/mL) and 150 mL xylazine (20 mg/mL)] and maintained for the lengths of time indicated below, before collecting blood via cardiac puncture. While still under deep anesthesia, blood samples were collected in heparinized tubes at 0 min, 30 min, 60 min, 180 min, and 330 min (n = 3 C57BL/6J mice for each time point) following treatment with the TD curcumin gel formulation. Blood collection tubes were centrifuged at 8,000 x g (12,000 rpm) for 10 min to pellet the cells. The supernatants (plasma) were transferred into fresh vials, and pelleted blood cells were kept in the original collection tube; plasma and blood cells were flash-frozen in liquid N_2_ (nitrogen) and stored at −80 °C until curcumin extraction and measurement.

**Extraction of curcumin from plasma**

The volume of recovered plasma from each mouse was measured using a micro-pipet. To the measured plasma, 100% acetonitrile (ACN, analytical grade or higher) was added to a final ACN concentration of 70% (v/v). Depicting the volume of ACN to be added as "X", the following equation was used to solve for X (mL):

$\frac{X}{X+P}=0.7$, where P is the volume of plasma in mL.

The tube was vigorously vortexed for 5 min and then centrifuged at 8,000 x g (12,000 rpm) for 10 min at room temperature. The supernatant was transferred to a fresh vial kept on ice. To the pellet, 500 µL of 70% ACN in dH_2_O was added and vortexed vigorously for 5 min followed by centrifugation at 8,000 x g (12,000 rpm) for 10 min. After centrifugation, the new supernatant was pooled with the first supernatant, and this process was repeated once more. The pooled supernatant was dried with a gentle stream of ultra-high purity, inert N_2_ gas, and the curcumin precipitate was stored at −80°C.

**Extraction of curcumin from the cell pellet**

Following centrifugation of the whole blood at 8,000 x g (12,000 rpm) for 10 min, the pellet containing all blood cells was flash-frozen and set aside for curcumin extraction. To each pellet, 500 µL 70% ACN in dH_2_O was added and the tube vortexed vigorously for 5 min. The sample was centrifuged at 8,000 x g (12,000 rpm) for 10 min at room temperature. The supernatant was transferred into a fresh vial. Extraction of the pellet and centrifugation was repeated twice, as described for plasma extraction; supernatants from each repetition were pooled. The combined supernatant was dried with a gentle stream of ultra-high purity, inert N_2_ gas, and the curcumin precipitate was stored at −80°C.

**High-performance liquid chromatography (HPLC) quantification of curcumin in the extracts**

Using a Hamilton syringe, 30 μL of each pooled extract resuspended in ACN were injected into an HPLC analyzer (1100 Series, Agilent Technologies, Santa Clara, CA, USA) fitted with a C18 reverse-phase 4.6 x 250-mm column (SN # 01873216412104; PN # WAT106151) procured from Waters Corp (Milford, MA, USA). Each sample was eluted from the column using a 30–70% gradient of ACN in water with 0.1% trifluoroacetic acid (TFA). Using UV-absorbance detection of curcumin at 430 nm, the curcuminoids present in each extract were detected as a triplet in the eluate at a retention time of approximately 10-13 min. A standard linear curve for curcumin was also constructed by injecting increasing concentrations of curcumin (5, 15, 25, 50, 75, 100, 125, 175, and 250 picomoles, each in 30 mL of ACN), collecting the eluates, and then graphically plotting the total peak areas from all the standard curcumin samples. Next, the summed value obtained from the peak areas for the three curcuminoid peaks from each extract was fitted into the equation for the standard curve for curcumin to obtain the curcumin content of each extract.^5^ Using the measured volume of each plasma sample, the plasma concentration of curcumin was calculated.

**Behavioral testing**

Mice were acclimatized to the testing room and apparatus prior to testing, as previously described.^2,4^ Pain related behaviors were analyzed, starting with mechanical, followed by musculoskeletal, and lastly, cold hypersensitivity, with sufficient time between tests for rest. Baseline (BL) recordings took place prior to administration, and post-treatment recordings were obtained after 1 hour and 24 hours, and on days 4, 7, 10, 14, and 21, following the start of vehicle or TD curcumin applications (Fig. S1). The 1 hour and 24-hour recordings were performed after the first dose and subsequent recordings were made prior to the delivery of the next dose. Cold avoidance behaviors were recorded at BL and 14 and 21 days after the start of treatment.

*Mechanical hypersensitivity:* Paw withdrawal frequency (PWF) evoked by a 1.0 g (4.08 mN) calibrated von Frey (Semmes-Weinstein) monofilament (Stoelting Co., Wood Dale, IL, USA) was recorded for 10 repeated applications to the plantar surface of each hind paw.^4^ Only vigorous withdrawal behaviors were recorded. Higher PWF is suggestive of more mechanical hypersensitivity.

*Musculoskeletal hypersensitivity*: To evaluate deep tissue/musculoskeletal hypersensitivity, the tensile force of peak forelimb exertion (grip force) was measured using a computerized grip force meter (SA Maier Co., Milwaukee, WI, USA). During testing, each mouse was held by its tail and gently passed over a wire mesh grid and allowed to grip the wires with only their forepaws. The peak force exerted against the transducer was recorded in grams for 3 repetitions and averaged for each mouse. Grip force measurements were normalized to individual body weights in grams. A lower grip force is indicative of more musculoskeletal hypersensitivity.

*Cold hypersensitivity*: To test for cold sensitivity, mice were placed onto a cold plate (4°C; U.G.O. Basile Model 35100, Collegeville, PA, USA), and the number paw withdrawals was recorded over a 2 min as PWF.^4^ Higher PWF is suggestive of greater cold hypersensitivity. Measurements were recorded once for PWF cold because sickle mice are highly sensitive to cold and may not survive with excessive cold exposure on repeated measures.

*Cold avoidance test:* Cold avoidance was measured as previously described.^3^ Mice were allowed to move freely between two connected chambers maintained at 30°C for 5 min to acclimate to the chambers and detect location preference, then the mice were removed from the testing apparatus (Ugo Basile Model 35250). One of the chambers was then cooled to 23°C, while the other chamber was left at 30˚C, and mice were re-introduced to the apparatus. The amount of time spent in the warm (30°C) and cold (23°C) chambers were recorded for a testing period of 5 min. Less time spent in the cold chamber suggests non-evoked cold avoidance and cold hypersensitivity.

**Isolation and culture of murine dorsal root ganglion (DRG) neurons.** Mice were humanely euthanized using compressed medical-grade CO_2_. The DRG from all vertebral column levels were removed and cultured as described previously.^6^ The DRG were transferred into ice-cold Dulbecco’s phosphate buffer saline (DPBS; Thermo Fisher Scientific, Waltham, MA, USA), cleaned from connective tissue, and transferred to Hank's Balanced Salt Solution without Ca^2+^ and Mg^2+^ ions (HBSS; Thermo Fisher Scientific) in a tube on ice. DRG were then dissociated with papain (Sigma Aldrich, Germany) and collagenase type 2 (Sigma Aldrich)/ dispase type 2 (Sigma Aldrich) solution in HBSS for 10 min each at 37ºC with intermittent centrifugation and resuspension of the pellet. To inhibit the proteolytic activity of enzymes, the pellet was washed with complete media (Ham's F-12 Nutrient Mix, Gibco™, Waltham, MA) containing 10% FBS (Gibco™) and 1% penicillin/streptomycin (Thermo Fisher Scientific). Finally, a single cell suspension of DRG neurons was prepared by constant trituration using a fire-polished glass Pasteur pipette and cultured on glass coverslips coated with poly-D-lysine (Sigma Aldrich) and mouse laminin (Thermo Fisher Scientific), placed inside 6-well cell culture plates.

**Sholl analysis for neurites of DRG neurons**

Primary DRG neurons were treated with either vehicle for TD curcumin, 1 ng/ml TNF-α (T) + 40 µM Hemin (H), 100 µM curcumin, or T+H+100 µM curcumin for 4 or 20h at 37˚C and immunostained with 1˚ rabbit anti-TUJ1/β3-Tubulin (1:200, # 5568, Cell Signaling Technology, Danvers, MA) and Cy3 AffiniPure donkey anti-rabbit 2˚ Ab (1:500, #711-165-152, Jackson ImmunoResearch, West Grove, PA). Combining 9 tile scans of 0.222 µm X 0.222 µm fields of view (arranged 3 X 3) with Z-stacks of 10 X 0.5 µm images were acquired on a laser scanning confocal microscope (Zeiss LSM 900, Carl Zeiss AG) using a plan-apochromat 20x/NA: 0.8 M27 objective lens and tile feature to acquire the images of individual soma and neurites. Sholl analysis was performed to quantify neurites using the Simple Neurite Tracer plugin for FIJI (http://fiji.sc/Welcome, ImageJ, NIH). Starting at 20 μm from the center point of the soma, concentric circles were constructed 20 μm apart, and neurite intersections were plotted against their radial distance from the soma. N=6-9/condition; ~3 mo old male HbSS-BERK sickle mice primary DRG neurons.^7,8^

**Body and organ weight**

The body weights of mice were evaluated at BL and at day 21 of treatment. At the study endpoint (day 21), mice were euthanized in accordance with IACUC regulations using compressed medical-grade CO_2_. Blood and organs (brain, heart, lungs, spleen, kidneys, and liver) were collected immediately after euthanasia, and the organs were weighed and then fixed in 10% buffered formalin for paraffin embedding and subsequent analysis.

**Assessment of organ pathology**

Liver and spleen sections of 4 µm thickness were stained with standard hematoxylin and eosin (H&E) or commercial iron stain (AR15892, Agilent Technologies). The stained specimens were observed under an Olympus Microscope BH-2 at 400x magnification to observe hepatic sinusoidal and splenic congestion (# per field), hepatic infarcts (% of fields), and hepatic and splenic iron deposits (# per field). Events were counted in a double-blind manner in 20 fields using 4 sections per slide.^9^

**Analysis of hematological parameters**

Whole blood was obtained following compressed CO_2_ euthanasia by cardiac puncture and was combined with 100 mM EDTA pH 7.5 at a 2:1 ratio (blood:EDTA) and immediately analyzed for hematocrit, total Hb, and complete blood counts using Animal Blood Counter (abc Plus, Scilvet, Viernheim, Germany). The remaining blood was smeared onto clean glass microscope slides and stained with reticulocyte stain (#R4132, Sigma Aldrich, Germany) to count the number of reticulocytes.^10^

**Lactate dehydrogenase (LDH) assay**

LDH activity was measured in plasma samples by a colorimetric assay according to the manufacturer’s instructions (MAK066, Sigma Aldrich, St. Louis, MO, USA).^11^ The absorbance was measured at 450 nm using a SpectraMax M3 plate reader (Molecular Devices, San Jose, CA, USA). Each specimen was run in duplicate with suitable negative and positive controls. LDH activity is reported as milliunit/mL. One unit of LDH activity is defined as the amount of enzyme that catalyzes the conversion of lactate into pyruvate to generate 1.0 µmole of NADH per minute at 37˚C.

**Measurement of basal adenosine triphosphate (ATP) level**

Intracellular ATP levels in red blood cells (RBCs) obtained after 21 days of curcumin or vehicle treatment from sickle mice were measured using a colorimetric ATP-assay kit (Sigma-Aldrich, St. Louis, MO, USA) as described.^12^ Briefly, RBCs were washed in Phosphate buffer saline (PBS; Thermo Fisher Scientific) after collection, and then 100 µl of packed RBCs were resuspended in PBS containing 1% glucose, 170 mg/L adenine, and 5 g/L mannitol for the ATP measurement. ATP concentration was determined by glycerol phosphorylation, which yields a colorimetric product proportional to the amount of ATP present, following the manufacturer’s instruction. Absorbance was measured at 570 nm using a BioTek Synergy HTX microplate reader (Agilent, Santa Clara, CA, USA).

**Estimation of protein carbonylation and lipid hydroperoxide formation**

As a measure of intracellular protein oxidation in frozen RBC lysates, protein carbonyl content was assessed by a dinitrophenyl hydrazine (DNPH) based assay kit (ab126287, Abcam, Cambridge, MA, USA).^13^ In these experiments, carbonyl groups in protein side chains are derivatized to DNP-hydrazone following reaction with DNPH. The absorbance of DNP hydrazones formed in this reaction was measured at 375 nm using a BioTek Synergy HTX microplate reader (Agilent, Santa Clara, CA, USA).

Lipid hydroperoxide levels were measured in plasma using a commercially available kit (#705002, Cayman Chemical Co., Ann Arbor, MI).^14^ Briefly, lipid hydroperoxides were extracted from 500 µl of plasma using a methanol based-extraction solution and chloroform. Deproteinated methanol-chloroform extract solution containing lipid hydroperoxides was measured by incubating with a chromogenic substrate as indicated in the manufacturer’s instructions. The absorbance was measured at 500 nm by a BioTek Synergy HTX microplate reader (Agilent, Santa Clara, CA, USA). Extracts without chromogenic substrate were kept to be used as blanks to eliminate the possibility of curcumin interference.

**Estimation of serum amyloid-P (SAP) in plasma**

SAP was estimated in plasma using an enzyme linked immunosorbent assay (ELISA) as described by the manufacturer (80660; Crystal Chem, Elk Grove Village, IL, USA) and as described.^3^ The absorbance for SAP assay was measured at 450 nm using a SpectraMax M3 plate reader. All analyses were run in duplicate with suitable negative and positive controls as described previously.^3^

**Mast cell analysis**

MCs in sections of mid-dorsal skin biopsies were stained with toluidine blue. In brief, the toluidine blue stain was prepared by dissolving 0.25 g toluidine blue (#01804, Chem-Impex International, Wood Dale, IL, USA) in 35 mL distilled water, 15 mL ethanol 100%, and 1 mL 1N HCl. After deparaffinization, the skin sections were incubated in toluidine blue for 1 min at room temperature, washed with dH_2_O, and air-dried. The stained specimens were observed under an Olympus Microscope BH-2 at 400x magnification to count MCs, recognized by red-purple metachromatic staining color on a blue background. MCs were counted in 20 fields, 4 sections per slide/mouse, and expressed as total MC number, number of degranulating MCs, and percentage of degranulating cells. Degranulating MCs are defined as cells associated with ≥ 8 granules outside the cell membrane, as described previously.^3,10^

**Skin releasate**

The dorsal skin of the mice was shaved the day before euthanasia. Mid-dorsal skin punch biopsies of 4 mm diameter were collected after euthanasia and washed in sterile phenol free Dulbecco's Modified Eagle Medium (DMEM; Gibco™) and incubated for 24 hours at 37°C in DMEM with 10,000 units penicillin/streptomycin as described.^6^ The conditioned medium was flash-frozen in liquid N_2_ and stored at -80°C until analysis.

**Cytokine array**

Cytokines were quantified in skin releasate using a microplate-based Q-Plex^TM^ microarray technology (Sample Testing Services of Quansys Biosciences Inc., Logan, UT, USA) as described.^10^ The Q-Plex^TM^ microarray uses traditional sandwich ELISA procedures on a microscale to simultaneously measure multiple cytokines included in our study - interleukins 1 alpha, 1 beta, 2-6, 10, 12, and 17 (IL-1α, IL-1β, IL-2, IL-3, IL-4, IL-5, IL-6, IL-10, IL-12, IL-17), monocyte chemoattractant protein 1 (MCP-1), interferon-gamma (IFNγ), tumor necrosis factor alpha (TNF-α), macrophage inflammatory protein-1 alpha (MIP-1α), granulocyte-macrophage colony-stimulating factor (GM-CSF), and regulated on activation, normal T-cell expressed and secreted protein (RANTES) were analyzed. Total protein estimation in the conditioned medium was performed for normalization using the Pierce Protein Quantitation Assay (#PI22662, Thermo Fisher Scientific).

**Statistical Analysis**

Data is shown as mean ± SEM and analyzed with one-way or two-way ANOVA with Tukey's or Dunnett’s multiple comparisons post hoc test or unpaired two-tailed T-test (Prism9, GraphPad, Boston, MA, USA) as described with each figure. Comparisons with P-values <0.05 are considered statistically significant.

**SI References**

1. Pászty C, Brion CM, Manci E, Witkowska HE, Stevens ME, Mohandas N, Rubin EM. Transgenic knockout mice with exclusively human sickle hemoglobin and sickle cell disease. Science 1997;278(5339):876-8.

2. Sagi V, Song-Naba WL, Benson BA, Joshi SS, Gupta K. Mouse Models of Pain in Sickle Cell Disease. Current Protocols in Neuroscience 2018;85(1):e54.

3. Cherukury H, Argueta DA, Garcia NR, Fouda R, Kiven SB, Lei J, Sagi V, Velasco GJ, Avalos B, DiPatrizio NV and others. Cannabidiol attenuates hyperalgesia in a mouse model of sickle cell disease. Blood 2023;141(2):203-08.

4. Kohli DR, Li Y, Khasabov SG, Gupta P, Kehl LJ, Ericson ME, Nguyen J, Gupta V, Hebbel RP, Simone DA and others. Pain-related behaviors and neurochemical alterations in mice expressing sickle hemoglobin: modulation by cannabinoids. Blood 2010;116(3):456-465.

5. Draganski A, Tar MT, Villegas G, Friedman JM, Davies KP. Topically Applied Curcumin-Loaded Nanoparticles Treat Erectile Dysfunction in a Rat Model of Type-2 Diabetes. J Sex Med 2018;15(5):645-653.

6. Khasabova IA, Uhelski M, Khasabov S, Gupta K, Seybold VS, Simone DA. Sensitization of nociceptors by prostaglandin E2-glycerol contributes to hyperalgesia in mice with sickle cell disease. Blood 2019;133(18):1989-1998.

7. Long BL, Li H, Mahadevan A, Tang T, Balotin K, Grandel N, Soto J, Wong SY, Abrego A, Li S and others. GAIN: A graphical method to automatically analyze individual neurite outgrowth. J Neurosci Methods 2017;283:62-71.

8. Chittajallu R, Wester JC, Craig MT, Barksdale E, Yuan XQ, Akgul G, Fang C, Collins D, Hunt S, Pelkey KA and others. Afferent specific role of NMDA receptors for the circuit integration of hippocampal neurogliaform cells. Nat Commun 2017;8(1):152.

9. Sargur Madabushi S, Fouda R, Ghimire H, Abdelhamid AMH, Lim JE, Vishwasrao P, Kiven S, Brooks J, Zuro D, Rosenthal J and others. Development and characterization of a preclinical total marrow irradiation conditioning-based bone marrow transplant model for sickle cell disease. Front Oncol 2022;12:969429.

10. Vincent L, Vang D, Nguyen J, Gupta M, Luk K, Ericson ME, Simone DA, Gupta K. Mast cell activation contributes to sickle cell pathobiology and pain in mice. Blood 2013;122(11):1853-1862.

11. Belcher JD, Chen C, Nguyen J, Zhang P, Abdulla F, Nguyen P, Killeen T, Xu P, O'Sullivan G, Nath KA and others. Control of Oxidative Stress and Inflammation in Sickle Cell Disease with the Nrf2 Activator Dimethyl Fumarate. Antioxid Redox Signal 2017;26(14):748-762.

12. Quezado ZMN, Kamimura S, Smith M, Wang X, Heaven MR, Jana S, Vogel S, Zerfas P, Combs CA, Almeida LEF and others. Mitapivat increases ATP and decreases oxidative stress and erythrocyte mitochondria retention in a SCD mouse model. Blood Cells Mol Dis 2022;95:102660.

13. Jana S, Kassa T, Wood F, Hicks W, Alayash AI. Changes in hemoglobin oxidation and band 3 during blood storage impact oxygen sensing and mitochondrial bioenergetic pathways in the human pulmonary arterial endothelial cell model. Front Physiol 2023;14:1278763.

14. Khaibullina A, Almeida LEF, Kamimura S, Zerfas PM, Smith ML, Vogel S, Wakim P, Vasconcelos OM, Quezado MM, Horkayne-Szakaly I and others. Sickle cell disease mice have cerebral oxidative stress and vascular and white matter abnormalities. Blood Cells Mol Dis 2021;86:102493.

**
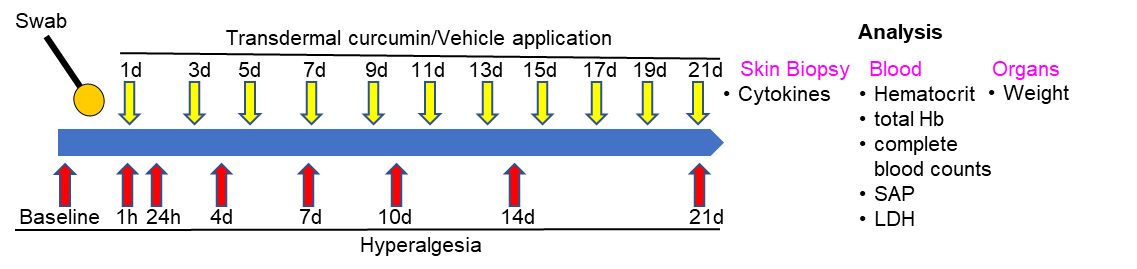
**

**Supplemental Figure S1. Study Design.** TD curcumin gel or vehicle were applied topically/transdermally on days, 1, 3, 5, 7, 9, 11, 13, 15, 17, 19, and 21 as indicated by yellow arrows. Baseline (BL) mechanical, cold and musculoskeletal hypersensitivity were measured before application, and after application at 1 hour and 24 hour and on days 4, 7, 10, 14, and 21 as indicated by red arrows. Cold avoidance test was done at BL and days 14 and 21. Mice were euthanized at the end of ~ 3 weeks and complete blood counts (CBC), plasma and tissues (skin, brain, heart, lungs, spleen, kidneys, liver) were collected.

**
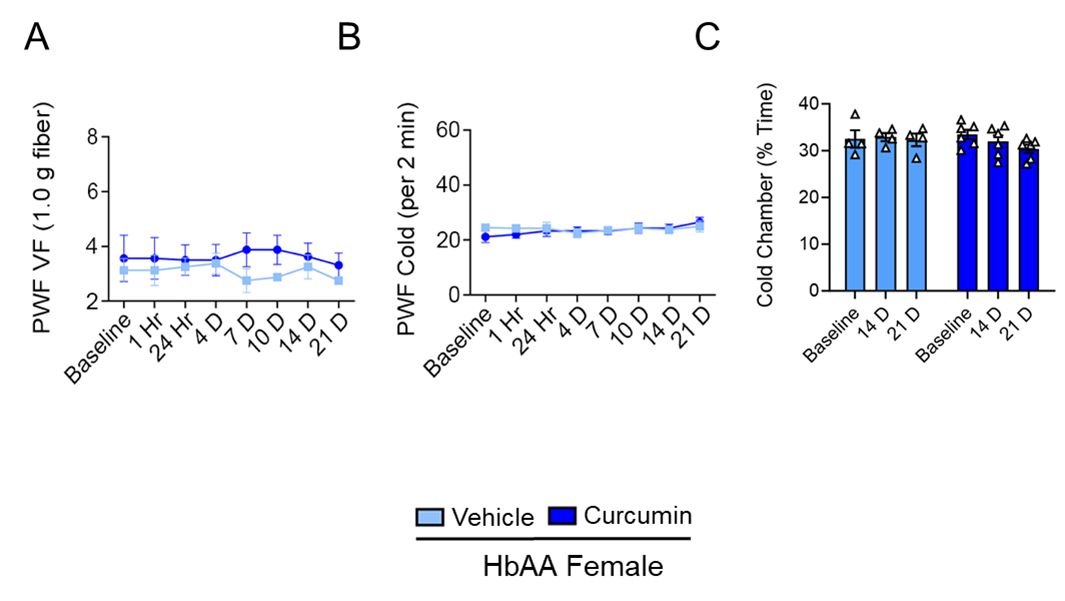
**

**Supplemental Figure S2. TD curcumin has no effect on pain-like behaviors in female HbAA mice.** Female HbAA mice were treated with vehicle or TD curcumin for 3 weeks on alternate days by gently rubbing the gel on the abdomen. Behavioral measures were obtained at baseline, and on days 1, 4, 7, 10, 14, and 21 after start of treatment schedule. TD curcumin showed no significant change in (A-B) mechanical and cold hypersensitivity indicated by no significant changes in PWF in response to von Frey monofilament (1.0 g fiber) application or 4°C cold plate exposure, respectively. TD curcumin did not alter (C) non-evoked cold avoidance, indicated by similar time spent in a cold chamber. Data shown as mean ± SEM. Age: Vehicle 5.40 ± 0.23 and TD Curcumin 5.37 ± 0.41 months. Analyzed with two-way ANOVA with Tukey's multiple comparisons test. Not significant P>.05. N=4-6. Abbreviations: PWF, paw withdrawal frequency; TD, transdermal; VF, von Frey.

**
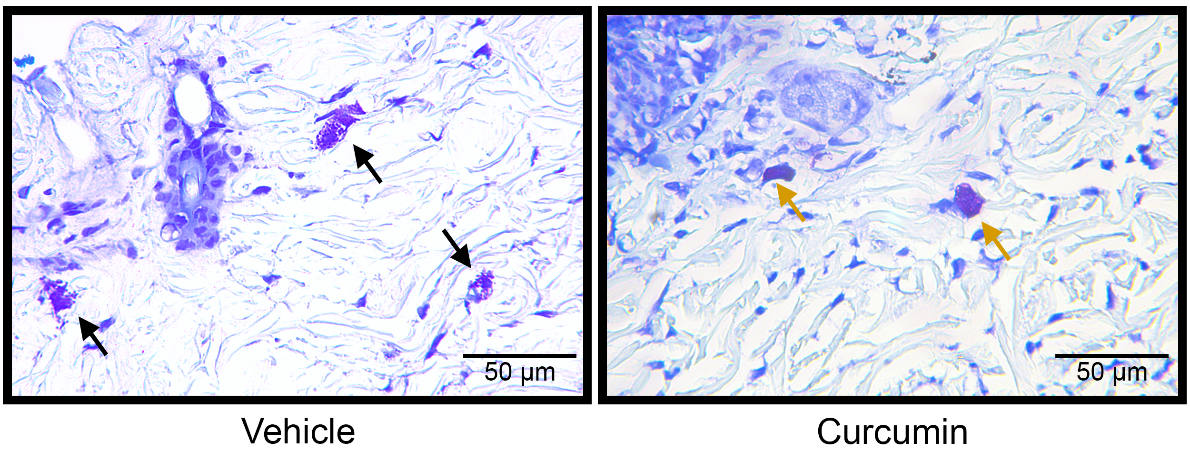
**

**Supplemental Figure S3. TD curcumin reduces mast cell degranulation in the mid-dorsal skin of male HbSS mice.** Male HbSS mice were treated with vehicle or TD curcumin for 3 weeks on alternate days by gently rubbing the gel on the abdomen. Mast cells in 4 µm skin sections were stained with toluidine blue and recognized by red-purple metachromatic staining color on a blue background. Mast cells were counted in 20 fields, 4 sections per mouse. Degranulating mast cells are defined as cells associated with ≥ 8 granules outside the cell membrane. Degranulating mast cells indicated with black arrows and intact mast cells indicated with yellow arrows. Representative images from N=4-6 mice per condition. Age: Vehicle 5.64 ± 0.89 and Curcumin 5.93 ± 1.13 months

**
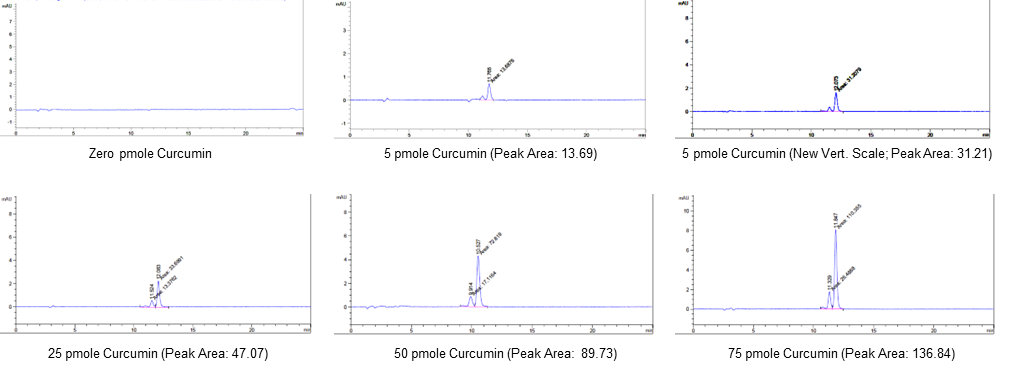
**

**Supplemental Figure S4. HPLC Profiles from Calibration Curves for Curcumin Recorded Using Absorbance Maximum at 430 nm.** A commercially available curcuminoid sample from Sigma containing 6% of bis-demethoxy-curcumin, 17% mono-dimethoxy-curcumin, and 77% curcumin was dissolved in 70% Acetonitrile (ACN) in water to prepare a standard solution. The Standard solution was serially diluted to obtain solutions containing 5, 15, 25, 50, 75, 100, 125, 175, and 250 pmole of curcumin in 30 microliters of 70% ACN. Each of these solutions was injected into a C18 HPLC column and eluted using a gradient of 30-90% ACN also containing 0.1% TFA. The eluted curcuminoids were detected using absorbance at 430 nm. Five sample eluate profiles of 5, 15, 25, 50, 75 pmole of curcuminoids are shown in the next slide. The areas under the three typical absorbance peaks in each eluate corresponding to 6% of bis-demethoxy-curcumin, 17% mono-dimethoxy-curcumin, and 77% curcumin are added and plotted against the pmole masses of curcuminoids to obtain a straight line (calibration curve). The profile obtained from 5 pmole of curcuminoids establishes the sensitivity of our method. The extracts of plasma and tissue samples containing curcuminoids are similarly eluted through the HPLC column and the total area under the triplet peak obtained is compared with the calibration curve to obtain a quantitative measure of curcuminoids

**Supplementary Table S1. ANOVA Results of HbSS Male and Female Pain-Related Behaviors.**

| **Mechanical Hypersensitivity Male HbSS Fig. 2A Two-way ANOVA** | | | *Alpha* | *0.05* |  |
| --- | --- | --- | --- | --- | --- |
| *Source of Variation* | *% of total variation* | *P value* | *P value summary* | *Significant?* |  |
| *Interaction* | 18.23 | 0.0001 | *** | Yes |  |
| *Time* | 2.416 | 0.7256 | ns | No |  |
| *Treatment* | 21.98 | < 0.0001 | **** | Yes |  |
| *ANOVA table* | *Sum Squared (SS)* | *Degrees of Freedom (DF)* | *Mean Squared (MS)* | *F (DFn, DFd)* | *P value* |
| *Interaction* | 17.64 | 7 | 2.52 | F (7, 104) = 4.796 | P = 0.0001 |
| *Time* | 2.337 | 7 | 0.3338 | F (7, 104) = 0.6355 | P = 0.7256 |
| *Treatment* | 21.26 | 1 | 21.26 | F (1, 104) = 40.47 | P < 0.0001 |
| *Residual* | 54.63 | 104 | 0.5253 |  |  |
| **Mechanical Hypersensitivity Female HbSS Fig. 2B Two-way ANOVA** | | | *Alpha* | *0.05* |  |
| *Source of Variation* | *% of total variation* | *P value* | *P value summary* | *Significant?* |  |
| *Interaction* | 17.00 | 0.0005 | *** | Yes |  |
| *Time* | 1.605 | 0.9004 | ns | No |  |
| *Treatment* | 27.40 | <0.0001 | **** | Yes |  |
| *ANOVA table* | *Sum Squared (SS)* | *Degrees of Freedom (DF)* | *Mean Squared (MS)* | *F (DFn, DFd)* | *P value* |
| *Interaction* | 15.46 | 7 | 2.209 | F (7, 88) = 4.228 | P=0.0005 |
| *Time* | 1.460 | 7 | 0.2085 | F (7, 88) = 0.3992 | P=0.9004 |
| *Treatment* | 24.92 | 1 | 24.92 | F (1, 88) = 47.72 | P<0.0001 |
| *Residual* | 45.96 | 88 | 0.5223 |  |  |
| **Cold Hypersensitivity Male HbSS Fig. 2C Two-way ANOVA** | | | *Alpha* | *0.05* |  |
| *Source of Variation* | *% of total variation* | *P value* | *P value summary* | *Significant?* |  |
| *Interaction* | 17.73 | 0.0011 | ** | Yes |  |
| *Time* | 10.31 | 0.041 | * | Yes |  |
| *Treatment* | 0.06128 | 0.7634 | ns | No |  |
| *ANOVA table* | *Sum Squared (SS)* | *Degrees of Freedom (DF)* | *Mean Squared (MS)* | *F (DFn, DFd)* | *P value* |
| *Interaction* | 1740 | 7 | 248.6 | F (7, 104) = 3.765 | P = 0.0011 |
| *Time* | 1012 | 7 | 144.5 | F (7, 104) = 2.190 | P = 0.0410 |
| *Treatment* | 6.012 | 1 | 6.012 | F (1, 104) = 0.09108 | P = 0.7634 |
| *Residual* | 6865 | 104 | 66.01 |  |  |
| **Cold Hypersensitivity Female HbSS Fig. 2D Two-way ANOVA** | | | *Alpha* | *0.05* |  |
| *Source of Variation* | *% of total variation* | *P value* | *P value summary* | *Significant?* |  |
| *Interaction* | 23.01 | <0.0001 | **** | Yes |  |
| *Time* | 13.77 | 0.0022 | ** | Yes |  |
| *Treatment* | 21.95 | <0.0001 | **** | Yes |  |
| *ANOVA table* | *Sum Squared (SS)* | *Degrees of Freedom (DF)* | *Mean Squared (MS)* | *F (DFn, DFd)* | *P value* |
| *Interaction* | 133.1 | 2 | 66.57 | F (2, 33) = 12.37 | P<0.0001 |
| *Time* | 79.67 | 2 | 39.84 | F (2, 33) = 7.401 | P=0.0022 |
| *Treatment* | 127.0 | 1 | 127.0 | F (1, 33) = 23.60 | P<0.0001 |
| *Residual* | 177.6 | 33 | 5.383 |  |  |
| **Cold Avoidance Male HbSS Fig. 2E Two-way ANOVA** | | | *Alpha* | *0.05* |  |
| *Source of Variation* | *% of total variation* | *P value* | *P value summary* | *Significant?* |  |
| *Interaction* | 26.08 | < 0.0001 | **** | Yes |  |
| *Time* | 12.92 | 0.0016 | ** | Yes |  |
| *Treatment* | 25.26 | < 0.0001 | **** | Yes |  |
| *ANOVA table* | *Sum Squared (SS)* | *Degrees of Freedom (DF)* | *Mean Squared (MS)* | *F (DFn, DFd)* | *P value* |
| *Interaction* | 173.7 | 2 | 86.85 | F (2, 39) = 15.36 | P < 0.0001 |
| *Time* | 86.08 | 2 | 43.04 | F (2, 39) = 7.610 | P = 0.0016 |
| *Treatment* | 168.3 | 1 | 168.3 | F (1, 39) = 29.76 | P < 0.0001 |
| *Residual* | 220.6 | 39 | 5.656 |  |  |
| **Cold Avoidance Female HbSS Fig. 2F Two-way ANOVA** | | | *Alpha* | *0.05* |  |
| *Source of Variation* | *% of total variation* | *P value* | *P value summary* | *Significant?* |  |
| *Interaction* | 23.01 | <0.0001 | **** | Yes |  |
| *Time* | 13.77 | 0.0022 | ** | Yes |  |
| *Treatment* | 21.95 | <0.0001 | **** | Yes |  |
| *ANOVA table* | *Sum Squared (SS)* | *Degrees of Freedom (DF)* | *Mean Squared (MS)* | *F (DFn, DFd)* | *P value* |
| *Interaction* | 133.1 | 2 | 66.57 | F (2, 33) = 12.37 | P<0.0001 |
| *Time* | 79.67 | 2 | 39.84 | F (2, 33) = 7.401 | P=0.0022 |
| *Treatment* | 127.0 | 1 | 127.0 | F (1, 33) = 23.60 | P<0.0001 |
| *Residual* | 177.6 | 33 | 5.383 |  |  |
